# Focused Ultrasound Mediated Opening of the Blood-Brain Barrier for Neurodegenerative Diseases

**DOI:** 10.3389/fneur.2021.749047

**Published:** 2021-11-04

**Authors:** Paul S. Fishman, Jonathan M. Fischell

**Affiliations:** Department of Neurology, University of Maryland School of Medicine, Baltimore, MD, United States

**Keywords:** FUS, blood-brain barrier, Alzheimer's disease, Parkinson's disease, focused ultrasound (MRgFUS)

## Abstract

The blood brain barrier (BBB) is an obstacle for the delivery of potential molecular therapies for neurodegenerative diseases such as Parkinson's disease (PD), Alzheimer's disease (AD), and amyotrophic lateral sclerosis (ALS). Although there has been a proliferation of potential disease modifying therapies for these progressive conditions, strategies to deliver these large agents remain limited. High intensity MRI guided focused ultrasound has already been FDA approved to lesion brain targets to treat movement disorders, while lower intensity pulsed ultrasound coupled with microbubbles commonly used as contrast agents can create transient safe opening of the BBB. Pre-clinical studies have successfully delivered growth factors, antibodies, genes, viral vectors, and nanoparticles in rodent models of AD and PD. Recent small clinical trials support the safety and feasibility of this strategy in these vulnerable patients. Further study is needed to establish safety as MRI guided BBB opening is used to enhance the delivery of newly developed molecular therapies.

In spite of major gains in the understanding of the biology of neurodegenerative disease such as Alzheimer's disease (AD), Parkinson's disease (PD), amyotrophic lateral sclerosis (ALS), and Huntington's disease, they remain progressively disabling and deadly conditions. Although many agents have provided neuroprotection in cellular and animal models of these conditions, none has resulted in clinically meaningful modification of their progressively worsening natural course. The need for effective disease modifying therapy (DMT) is so dire that agents with likely marginal benefit such as the recently FDA approved aducanumab for AD generate great public interest (1).

Although the factors responsible for the widespread failures of promising agents to translate into clinically effective DMTs are complex, a role for poor brain bioavailability has been suggested (2). This is particularly true for the growing pharmacopeia of molecular therapies including growth factors, enzymes, monoclonal antibodies, and genetic material, all too large to cross the specialized endothelia that compose the blood-brain barrier (BBB). Unfortunately, the intensity of research progress in the development of molecular therapies has greatly outpaced the development of strategies for their delivery to brain.

Contemporary clinical trials of gene therapy for neurodegenerative disease continue to rely on invasive methods such intracerebral infusion (3, 4). The most well-explored strategy to allow large molecules to cross the BBB has been the creation of hybrid molecules that contain a domain the binds to brain endothelial membrane transport receptors such as the transferrin and insulin receptors (5). These “trojan horse” therapeutics have begun to enter clinical trials (6). There has been a resurgence in interest in intra-arterial infusion of hyperosmolar solutions of mannitol to open the BBB, a method initially developed in the 1980's (7, 8).

The newest strategy to enhance delivery of therapeutics from blood to brain is to use focused ultrasound (FUS). The specialized endothelia of the brain have continuous tight junctions that form the BBB, limiting the movement of large molecules from the bloodstream into brain. Studies by Hynynen, McDannold, and colleagues (9–12) initially demonstrated that FUS applied during the circulation of microbubble suspensions (FDA-approved ultrasound contrast agents) can create a transient and safe disruption of the BBB, which can be targeted to a specific brain region using MRI. This allows large therapeutics to enter the brain from the systemic circulation including antibodies, growth factors, nanoparticles, nucleic acids, viral vectors, and even cells (13–19). Using pulsed ultrasound at a much lower intensity than the continuous ultrasound used for brain tissue ablation, the microbubbles undergo oscillations of expansion and contraction that cause transient separation of endothelial tight junctions—the basis for the BBB (20, 21). The procedure can create transient (hours) opening of the BBB, sufficient to allow extravasation of large therapeutics without pathology or entry of blood components (22, 23).

Delivery of large therapeutics across the BBB with any strategy has been limited by the inefficiency of the transfer where accumulation of 1–2% of the total blood injectate in the brain is a true accomplishment (5). Within safe parameters, BBB opening may last only a few hours, and the amount of the therapeutic entering brain is usually much less. Studies of molecular therapies usually find that <0.1% of the injected agent can be detected in the sonicated region of brain after MRgFUS-mediated opening of the BBB (24).

The first application of this strategy was in brain tumor therapy where in preclinical models of brain metastatic breast cancer, FUS-mediated BBB opening substantially improved the efficacy of the antihuman epidermal growth factor 2 monoclonal antibody trastuzumab (24). Clinical trials of FUS opening to enhance chemotherapy of brain tumors are currently in progress (25).

For neurodegenerative diseases, studies using MRI guided FUS (MRgFUS) have enhanced delivery of several potential DMTs including genes in preclinical models of PD. The delivery of glial cell-derived neurotrophic factor (GDNF) and the related factor neurturin from the blood was improved in rodents with the use of this strategy (26, 27). Gene delivery of GDNF has been successful in restoring dopamine metabolism and reversing motor abnormalities in a toxin-induced rat model of PD (28). In an effort to improve the efficiency of delivery (a persistent problem with all blood to brain strategies), the plasmid was preloaded into the microbubbles to enhance its concentration in the region of FUS-mediated BBB opening.

Gene delivery for GDNF has also been successful with enhanced brain distribution using brain penetrant nanoparticles with FUS mediated BBB disruption (D) in the 6-OHDA rodent model of PD (29). Another strategy to improve efficiency of delivery of FUS mediated BBBD has been the use of viral vectors (19, 30). Gene delivery using an adeno associated viral (AAV) vector for GDNF was effective in ameliorating the sub-acute MPTP injection rodent model of PD (31). These studies demonstrate that FUS mediated BBBD can clearly be combined with other delivery methods in the goal of improving the efficiency of brain delivery while still minimizing invasiveness. In a highly novel approach, FUS enhanced the distribution of intranasally delivered BDNF. Although the mechanism of this enhancement is uncertain, this combination successfully improved mice exposed to MPTP (32). Similar mechanism may underlie FUS mediated improvement in distribution of therapeutics after convection-enhanced intracerebral injection, which is the current clinical standard for delivery of protein and gene therapy to the brain (33, 34).

More than 30 years after the initial clinical trials, cell-based therapy has yet to fulfill its promise as a DMT for neurodegenerative disease. As with gene therapy, clinical studies of stem cells for PD still rely on intraparenchymal injection (35, 36). In spite of their large size, rodent studies have demonstrated enhanced delivery of stem cells to brain from the blood, which may involve active mechanism such as chemoattraction and transcytosis across the brain endothelium (37). At this point, however, a beneficial effect of FUS enhanced stem cell delivery has yet to be demonstrated in an animal model of a neurodegenerative disease.

There is also a strong body of evidence from animal studies suggesting the potential benefit of BBBD in AD. These studies explored the possibility that BBBD could be a tool to accelerate the clearance of beta-amyloid from the brain—a major goal of many current AD experimental therapeutics. Studies in mouse models of AD have demonstrated both reduction in brain amyloid burden and behavioral improvement using BBBD coupled with either infused or endogenous anti-amyloid antibodies (18, 38). This reduction in amyloid burden and improved behavior occurred without evidence of brain hemorrhage, a known risk of FUS as well as a clear concern when considering the coexistence of amyloid angiopathy in AD with its significant risk of brain hemorrhage (39). One of the AD/amyloid animal studies demonstrated that moving the target of sonication through the brain (scanning FUS) could be a potential useful strategy for treatment of a large brain volume (40).

As with most antibodies, relatively little of an anti-amyloid antibody enters the brain from an IV injection [0.1% (41)]. However, these antibodies appear to have the capacity to accumulate in the AD brain, likely due to binding to brain amyloid (42). The mechanism by which anti-amyloid antibodies in the blood reduce brain amyloid burden without significant entry into brain remains controversial and how FUS potentiates amyloid clearance is under active investigation (43, 44). There is also concern that compromise of the BBB may also interfere with normal amyloid clearance from the brain (45).

FUS mediated BBB opening also appears to stimulate neurogenesis in the treated region, although once again the mechanism involved is uncertain (46, 47).

In spite of these promising pre-clinical rodent studies, the path to clinical trials of BBBD for neurodegenerative disease is not straightforward. All potential therapeutics utilized in the animal studies are experimental, without full FDA approval. A trial that combines a first of its kind study of an experimental delivery method such as FUS mediated BBBD with an experimental agent such as gene therapy would pose unknown risks to this fragile patient population that may be additive. This is particularly relevant to the combined use of BBBD for delivery of an anti-amyloid antibody where there is overlap in potential adverse effects and pathology. FUS mediated BBBD carries with it a risk of excessive disruption of the BBB resulting in brain edema and hemorrhage although neither pathology was observed in animal studies with transgenic AD mice or aged dogs with amyloid deposition (48). An increase in multiple inflammatory markers has been described in other animal studies of FUS mediated BBBD (49). Both brain edema and hemorrhage are also risk of treatment with monoclonal antibodies against amyloid for AD (50).

The possibility of additive and unknown risks with the combination of an experimental agent and an experimental delivery method influenced the design of the first clinical trial of FUS mediated BBBD in patients with AD. Supported by the pre-clinical studies, patient with mild to moderate AD underwent BBBD alone, without infusion of an anti-amyloid antibody (51). A frontal cortex location was chosen as the sonication target, reflecting the emphasis on safety in this first of kind study. Although a hippocampal target could be viewed as more clinically important, its deep location was felt to pose a greater risk if significant edema or bleeding were to occur. Patients who tolerated a relatively small volume of BBBD had the procedure repeated later targeting a larger volume. This study supported both the feasibility of opening the BBBD in AD patients (using gadolinium extravasation as an outcome measure) as well as its safety. No significant change in amyloid signal on PET scans or cognitive change was detected, although the study was not powered to assess these efficacy outcomes. There was no significant edema or bleeding among the five treated patients, although transient evidence of possible micro-hemorrhage was observed.

A subsequent study of six patients with AD targeting the hippocampus also supported the safety of FUS mediated BBBD (52). This group of patients also showed a modest reduction of amyloid related signal on 18 F-Florbetaben PET the after three rounds of BBBD over 6 months (53).

It should be noted that this is one of several setting where FUS mediated BBBD will need to be performed on a repeated basis. This will also increase an aspect of brain FUS that reduces patient satisfaction, that of complete shaving of the head, which contemporary neurosurgical procedures tend to avoid. With the recent FDA approval of aducanumab (Aduhelm), it is likely that this agent will be combined with FUS mediated BBBD in the near future. A recent study in an AD transgenic mouse has already assessed the combination of a murine aducanumab analog and BBBD using scanning FUS (54). As expected, FUS substantially increased the distribution and amount of this anti-amyloid antibody in the brain. It is encouraging that a potential safety outcome—brain microhemorrhages—were not increased in any of the treatment groups including combination treatment. Although the effect on clearance of amyloid from the brain varied with brain region (increased clearance with combination treatment of frontal cortex but not in the hippocampus), there was substantial improvement in performance on a spatial memory tasks only with combination treatment. Although the hypothesis is that this combined approach will be more effective than either modality alone for both removal of cerebral amyloid and clinical improvement, safety will remain the primary outcome measure in the initial studies.

A similar approach has been utilized in patients with PD dementia (55). Although PD has predominantly intracellular accumulation of pathologic forms of alpha synuclein, PD dementia is strongly associated with a combination of alpha synuclein and amyloid deposition (56). This recent study of five patients supports the safety of multiple repeated rounds of BBBD of a target region at the parieto-occipito-temporal junction. Although mild improvement in cognition was observed, no significant change in either amyloid or fluorodeoxy glucose by PET scan was observed.

The strategy of combining FUS mediated BBBD with an approved therapeutic is currently underway with a potential DMT for PD. There is a strong association between Gaucher's disease, a lysosomal storage disease caused by mutant forms of the enzyme glucocerebrosidase (GCase) with PD (57). Studies of the interaction of this enzyme with alpha synuclein have also supported GCase as a possible DMT for PD (58). A recombinant form of normal GCase has been an FDA approved therapy for Gaucher's disease for many years (59). As with all forms of enzyme replacement therapy, large molecular size prevents crossing the BBB with limited benefit for CNS forms of diseases associated with detective endogenous enzyme such as Gaucher's. A clinical trial where PD patients are infused intravenously with GCase at the same time as BBBD targeted to the basal ganglia is currently in progress (ClinicalTrials.gov identifier NCT04370665).

The safety of BBBD even in patients where a region with symptomatic neuronal dysfunction is directly targeted is supported by the first study of FUS mediated BBBD in patients with ALS (60). The four volunteers showed no worsening of their motor function after successful BBBD targeted to their motor cortex. The goal of this study as preparation to deliver molecular therapeutics to corticospinal neuronal cell bodies reflects a contemporary review of ALS which emphasizes both the physiologic importance of these “upper motorneurons” in motor symptoms as well as the “dying forward” nature of its pathogenesis that likely begins in the cell soma (61, 62).

These studies illustrate the potential of FUS mediated BBBD to enhance potential DMT for neurodegenerative disease. The initial clinical experience suggests that pulsed FUS coupled with microbubble infusion can result in safe, transient localized opening of the BBBD in patients with AD, PD, and ALS. Pre-clinical studies suggest that this form of BBBD may amplify the efficacy of circulating endogenous and exogenous molecules that are too large to cross the normal BBBD. As the safety of FUS mediated BBBD becomes more established, the opportunities for utilizing it to delivery experimental therapies show as gene therapies will develop. The combination of effective molecular agents and a safe strategy to enhance their delivery to brain such as FUS mediated BBB opening could accelerate the development of clinical useful disease modifying therapies for the million of patients suffering from progressive neurodegenerative diseases.

## Author Contributions

PF had primary responsibility for the review of the studies cited and writing of the paper. JF verified the accuracy of the representation of the cited literature. Both authors contributed to the article and approved the submitted version.

## Conflict of Interest

PF has received funding from Insightec which has funded some of the clinical studies cited. The remaining author declares that the research was conducted in the absence of any commercial or financial relationships that could be construed as a potential conflict of interest. The handling Editor declared a past co-authorship with one of the authors, PF.

## Publisher's Note

All claims expressed in this article are solely those of the authors and do not necessarily represent those of their affiliated organizations, or those of the publisher, the editors and the reviewers. Any product that may be evaluated in this article, or claim that may be made by its manufacturer, is not guaranteed or endorsed by the publisher.
